# Risk Assessment of Urban Rainstorm Disaster Based on Multi-Layer Weighted Principal Component Analysis: A Case Study of Nanjing, China

**DOI:** 10.3390/ijerph17155523

**Published:** 2020-07-30

**Authors:** Junfei Chen, Juan Ji, Huimin Wang, Menghua Deng, Cong Yu

**Affiliations:** 1 Business School, Hohai University, Nanjing 211100, China; jijuan26@hhu.edu.cn (J.J.); hmwang@hhu.edu.cn (H.W.); dengmh@hhu.edu.cn (M.D.); yucong9663@hhu.edu.cn (C.Y.); 2 Yangtze Institute for Conservation and Development, Hohai University, Nanjing 210098, China; 3 Research Institute of Jiangsu Yangtze River Conservation and High-Quality Development, Nanjing 210098, China; 4 State Key Laboratory of Hydrology-Water Resources and Hydraulic Engineering, Hohai University, Nanjing 210098, China

**Keywords:** multi-layer weighted, principal component analysis, urban rainstorm disaster, risk assessment

## Abstract

Nanjing city is taken as a case in this urban rainstorm disaster risk research. Using the data of meteorology and social-economy statistics of Nanjing area, the paper selected ten indicators to establish the risk assessment system of urban rainstorm disaster from the aspects of the vulnerability of hazard-affected body, the fragility of disaster-pregnant environment, and the danger of hazard factors. Multi-layer weighted principal component analysis (MLWPCA) is an extension of the principal component analysis (PCA). The MLWPCA is based on factor analysis for the division subsystem. Then the PCA is used to analyze the indicators in each subsystem and weighted to synthesize. ArcGIS is used to describe regional differences in the urban rainstorm disaster risk. Results show that the MLWPCA is more targeted and discriminatory than principal component analysis in the risk assessment of urban rainstorm disaster. Hazard-affected body and disaster-pregnant environment have greater impacts on the risk assessment of rainstorm disaster in Nanjing, but the influence of hazard factors is few. Spatially, there is a large gap in the rainstorm disaster risk in Nanjing. The areas with high-risk rainstorm disaster are mainly concentrated in the central part of Nanjing, and the areas with low-risk rainstorm disaster are in the south and north of the city.

## 1. Introduction

In the long course of human society, on the one hand, people use water for irrigation, power generation, and navigation. On the other hand, human beings are also affected by natural disasters such as flood, waterlogging, and rainstorm. According to the statistics of the International Emergency Disaster Database (EM-DAT), the rainstorm disaster is one of the most frequent disasters in the world, which brings huge risks and losses to the city [1]. With the rapid development of economy, high urbanization makes the loss of urban rainstorm disaster increase, and the loss of urban wealth and population concentration area is greater than in the past. In 2016, the Yangtze River Basin suffered the worst flood, influencing more than 100 million people in 31 provinces and cities in China and causing direct economic loss with more than 360 billion yuan [2]. During the nine years from 2010 to 2018, as many as 1016.791 million people were affected by the rain and flood disasters, and the direct economic loss reached 2235.928 billion yuan, as shown in Table 1.

From Table 1, it can be seen that urban rainstorm disaster not only does great harm to the safety of residents but also cause great economic losses in China. In order to reduce the losses caused by urban rainstorm disaster, many scholars have carried out research on the risk assessment of urban rainstorm disaster in recent years, specifically from three aspects: (1) Studying the formation mechanism of urban rainstorm disaster risk. Different understanding of the formation mechanism of urban rainstorm risk will inevitably lead to different expressions of disaster risk degree. Maskrey [3] proposed that natural disaster risk is the algebraic sum of risk and vulnerability, which reflects the essential characteristics of risk more comprehensively. In 1991, the United Nations [4] proposed that natural disaster risk is the product of risk and vulnerability. At present, most scholars believe that urban rainstorm risk is determined by three factors: risk, stability, and vulnerability. This is similar to the concept model of “rainstorm flood risk triangle” proposed by Crichton et al. [5]. Some researchers take disaster prevention and mitigation capacity [6] into account when analyzing the risk of urban rainstorm disaster. They think that the risk, stability, vulnerability, and disaster prevention and mitigation capacity are the results of the four factors. (2) Studying the evaluation methods. There are many rainstorm risk assessment methods according to the different index systems of urban rainstorm risk assessment. Probability statistical method based on historical disaster is an important method of risk assessment. Benito et al. [7] used the method of combining historical flood data with geology, history, statistics, and other disciplines to assess rainstorm risk. Grey relational analysis method [8,9], Hydro hydraulics method [10], complex network [11], expert analysis in conjunction with field survey and Analytical Hierarchy Process (AHP) [12], and the analytic hierarchy process [13] are widely used in the evolution process and risk assessment of urban rainstorm disasters. These methods have their advantages and disadvantages. Probability statistical method is based on historical disaster, the calculation is simple, and the background data such as topography and landform are not needed. However, this method also has its shortcomings. The quality of historical disaster data and the length of the cycle will directly affect the evaluation results. The grey correlation analysis method has a clear idea, simple and fast process, and is easy to program, but it is too subjective to determine the optimal value of each index. In hydro hydraulics method, it is difficult to obtain parameters because it is mainly applied to a small area and is difficult to be applied on a larger scale. Complex network method can avoid the error caused by subjective weighting, but it is not easy to explain the function and relationship of each parameter. AHP is clear in concept and easy to calculate and understand. The biggest drawback of this method is its strong subjectivity. Moreover, the MLWPCA method can avoid the phenomenon of subjective weighting, and has more pertinence and differentiation than the principal component analysis. (3) Studying risk assessment and regionalization. Mukhopadhyay et al. [14] established the time-space distribution model of precipitation based on the hydrodynamics theory and evaluated the flood risk of the piedmont alluvial plain terrain. Tawatchai et al. [15] evaluated the risk of flood disaster in southwest Bangladesh using self-built model. Mu et al. [16] performed rainstorm waterlogging disaster simulation, based on the advantages of remote sensing. Furthermore, an alternative integrated model was presented from hazard assessment, vulnerability assessment, and exposure analysis on the basis of meteorological, hydrographic, and other professional knowledge, combining Global Position System (GPS) and Geographical Information System (GIS). Some scholars [17,18,19,20,21,22] have built a rainstorm risk assessment model based on ArcGIS.

The existing research has obtained some theoretical and operational research results. However, in terms of practicability, the risk assessment of urban rainstorm disaster has obvious regional characteristics. It is necessary to select assessment factors according to local conditions. Factors are quantified and classified. Besides, historical data and expert experience are comprehensively used to improve the accuracy and speed of the evaluation. Nanjing, Jiangsu Province, is located in the lower reaches of the Yangtze River. The Yangtze River flows through the main urban area of Nanjing. Therefore, the rain and flood disaster in Nanjing is affected not only by its own precipitation but also by the upstream cities. On this foundation, thus, Nanjing city is taken as a case in this urban rainstorm disaster risk research. Through the comparative analysis of the two methods of PCA and MLWPCA, the paper describes the regional differences of rainstorm disaster risk in each district of Nanjing by ArcGIS. It provides a new method for risk assessment of rainstorm disaster in Nanjing to find out the cause of high risk of rainstorm disaster. This paper has important theoretical value and practical significance. At the same time, it also expands the methods in the field of risk assessment.

## 2. Study Area

**Figure 1:**
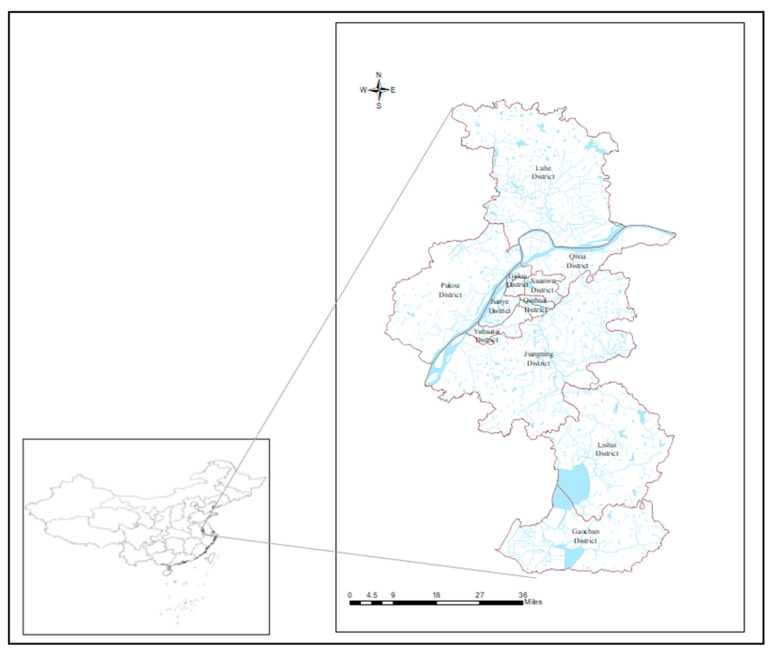
The location of 11 districts in Nanjing, China.

The research area is 11 administrative regions in Nanjing, Jiangsu Province, China. Six main urban areas are included such as Gulou District and Jianye District, with five suburbs such as Jiangning District, Pukou District, and Gaochun District (Figure 1). Nanjing is located in the east of China, the lower reaches the Yangtze River and the southwest of Jiangsu Province. It is not only a sub-provincial city in China but also the capital of Jiangsu Province. Its geographical coordinates are 31°14″ N-32°37″ N, 118°22″ E-119°14″ E. The landform of Nanjing is characterized by the mountains and hills, with an average elevation of 20–30 m. The water area accounts for more than 11%. There are Qinhuai River, Lishui River, and other rivers in the city. The water system is developed and has certain regulation and storage capacity for urban rainstorm. Nanjing enjoys a typical subtropical monsoon climate, significantly affected by the monsoon climate. The annual average rainfall is more than 1100mm, and the annual average rainfall days are about 120 days. The rainfall shows uneven distributions in time and space while the time distributions are mainly reflected. The rainfall is mainly concentrated in the flood season (May to September). The frequent urban rainstorm incidents in the flood season could easily cause urban rainstorm disasters.

With the process of urbanization, Nanjing, as a megacity in the Yangtze River Delta and East China, has achieved 82.5% urbanization rate by the end of 2018. Moreover, the problem of urban rainstorm disaster has also become increasingly prominent. In recent years, there have been large or small urban rainstorm disasters in Nanjing almost every year. For example, in June 2015, three consecutive days of torrential rain caused the inland river to overflow the dike. Then, the village was flooded and the water depth in some areas reached 2 m. In July 2016, Nanjing suffered serious rainstorm and flood disasters. By July 2019, Nanjing had to open the “the mode of watching the sea” every year. Therefore, it is very necessary to strengthen the research on the risk management of rainstorm disaster in Nanjing, which is of great significance to mitigate and prevent the risk of rainstorm disaster in Nanjing. 

## 3. Data and Methods

### 3.1. Date Sources

The research period is from May to September 2018 in 11 districts of Nanjing. Although the area of each administrative region in Nanjing is quite different, this paper focuses on the difference of risk distribution of rainstorm disaster in different administrative regions of Nanjing. Therefore, the minimum unit of the study area is set as the administrative region level. Through consulting “Nanjing Statistical Yearbook”, “Jiangsu Province Water Conservancy Yearbook”, “Nanjing Water Resources Bulletin”, “Nanjing Meteorological Monthly” and other data, as well as the field investigation and collections of the research team, the risk assessment index data of rainstorm disaster in the flood season (May in 2018 to September in 2018) in 11 sub-districts of Nanjing, are obtained. Among them, the data of population, GDP, and drainage network density are obtained from “Nanjing Statistical Yearbook”. The data of water area, 24-h maximum rainfall, total rainfall, and rainstorm days were obtained from “Jiangsu Province Water Conservancy Yearbook”, “Nanjing Water Resources Bulletin”, and “Nanjing Meteorological Monthly". The official website of each administrative region can obtain some data, including land area, building area, and percentage of vegetation coverage.

### 3.2. Selection of Evaluation Indicators 

Urban rainstorm disaster is the result of the combination of various natural and social factors. In the past, scholars mostly analyzed the risk of urban storm flood disaster from the vulnerability of hazard-affected body, the danger of hazard factors, the fragility of disaster-pregnant environment, and the ability of disaster prevention and mitigation [23,24,25,26,27,28]. Based on the research of these scholars, this paper considers that the vulnerability of the hazard-affected body has obvious correlation with the ability of disaster prevention and mitigation, that is, the higher the economic level and population quality of the disaster-bearing body, the stronger its ability of disaster prevention and mitigation. According to the establishment principle of index system, this paper constructs the risk assessment system of rainstorm disaster in Nanjing from three aspects: the vulnerability of hazard-affected body, the fragility of disaster-pregnant environment, and the danger of hazard factors [29]. Through literature research, the Three Gorges reservoir has effectively reduced the flood control pressure in the middle and lower reaches of the Yangtze River [30,31]. Nanjing is located in the lower reaches of the Yangtze River. The flood discharge is large but the flow rate is slow. Moreover, the Three Gorges reservoir has also intercepted some floods [32,33], which greatly reduces the risk of rainstorm disaster in Nanjing. Affected by the subtropical monsoon climate, Nanjing has a lot of precipitation in flood season. Therefore, this paper mainly studies the impact of internal factors on the risk of rainstorm disaster in Nanjing.

#### 3.2.1. The Vulnerability of Hazard-Affected Body

The greater vulnerability of hazard-affected body of urban rainstorm disaster indicates that the city’s ability to withstand rainstorm disasters is lower. The paper selects four assessment indicators of the vulnerability of hazard-affected body, including population density (person/km^2^), building density (%), subway station density (one/100 km^2^), and GDP of per land (billion yuan/km^2^). 

Population density refers to the ratio of the total population of a region to the administrative area of the region. The larger the population density is, the more people will be affected and the greater the loss will be. Building density refers to the density of buildings in a certain urban area. The higher the building density is, the greater the risk of urban rainstorm disaster is. Subway station density is the number of subway stations per unit area. It mainly reflects the degree of public transportation. The higher the subway station density is, the greater the possibility of damage after rainstorm, and the risk of urban rainstorm disaster are correspondingly high. GDP of per land can reflect the regional economic concentration and economic development level of a city. The higher the GDP of per land is, the greater the risk of urban rainstorm disaster is.

#### 3.2.2. The Fragility of Disaster-Pregnant Environment

Fragility refers to the fragility of disaster-pregnant environment to respond to urban rainstorm disaster and refers to the environmental impact factors that cause the risk of urban rainstorm disaster. The disaster-pregnant environment of urban rainstorm disaster can be divided into two categories: the natural environment that breeds urban rainstorm disasters and the social environment that breeds urban rainstorm disasters. The incubation environment of urban rainstorm disasters is formed by the interaction of many factors. The natural environment mainly includes the related indexes of topography and landform, and the social environment mainly includes the related indexes of drainage pipe network and road network. Therefore, this paper selects three indicators of vegetation coverage rate (%), percentage of water area (%), and drainage network density (km/km^2^) as the indicators of fragility of disaster-pregnant environment. 

Vegetation coverage rate represents the ratio of the vertical projection area of urban plants to the area of the administrative area. The higher the vegetation coverage rate is, the higher the greening degree is, and the stronger the water and soil conservation capacity and rainwater absorption capacity are, the lower the risk of rainstorm disaster is in this area. Percentage of water area refers to the proportion of water area in the region to the total area of the region. The higher the percentage of water area is, the stronger the water storage regulation capacity of the city is, and the risk of urban rainstorm disaster is correspondingly low. Drainage network density refers to the ratio of the total length of drainage pipelines to the area of built-up area in a certain area of a city, which indicates the density degree of regional drainage pipelines. The higher the density of the urban drainage network is, the stronger the drainage capacity of the city is. After the occurrence of the rainstorm disaster, the stronger the drainage capacity of the city is, the lower the risk of the urban rainstorm disaster is.

#### 3.2.3. The Danger of Hazard Factors

The danger refers to the danger of the disaster causing factors of urban rainwater and flood disaster. Generally speaking, the greater the danger of the disaster-causing factors of urban rainstorm disaster, the higher the risk level of urban rainstorm disaster. In this paper, 24-h maximum rainfall (mm), rainstorm days (days), and total rainfall (mm) are selected as risk assessment indicators of urban rainstorm disaster. 

24-h maximum rainfall refers to the maximum rainfall in one day in a month, which reflects the rainfall intensity in a short period of time in a certain area. Rainstorm days refer to the days when the rainfall exceeds 50 mm in a day in the study period. The index of monthly rainstorm days considers the influence of rainfall duration on urban rainstorm disaster. The more rainstorm days in the month are, the greater the possibility of water accumulation on the ground and the possibility of urban rainstorm disaster are. Total rainfall refers to the total amount of accumulated rainfall in a certain region during the study period. The greater the total rainfall in a certain period in a certain region is, the greater the possibility of urban rainstorm disaster is.

### 3.3. Research Methods 

#### 3.3.1. Index Standardization

To take 11 administrative units in Nanjing as research objects, 10 evaluation indexes are selected. Due to the inconsistent data dimension of each indicator, there are great differences between the data, which may have an unreasonable impact on the final results after standardized processing. In this paper, the method mentioned by Jiao [34] is adopted, that is, the combination of linear scaling method and the range transformation method, as follows:

When the indicator “bigger is better”, the upper limit effect measure can be used:

(1) $$C_{ik}=\frac{f_{ki}-f_{k1}}{f_{k2}-f_{k1}},$$

When the indicator “smaller is better”, the lower limit effect measurement can be used:

(2) $$\;C_{ik}=\frac{f_{k2}-f_{ki}}{f_{k2}-f_{k1}},$$

where $f_{ki}$ is any value in the indicator, $f_{k1}$ is the minimum value of the indicator in all modes, and $f_{k2}$ is the maximum value of the indicator in all models.

After the standardizing, a normalized matrix of order 11×10 was obtained. Then, based on the principal component analysis, the extended structural hierarchical principal component analysis method [35,36] was used to evaluate the risk of rainstorm disasters in Nanjing. The spatial differentiation of rainstorm risk levels in Nanjing is described by using ArcGIS (ESRI, RedLands, CA, USA).

#### 3.3.2. The Core Idea of PCA

The core idea of principal component analysis is to reduce the dimension of the original data, select the number of principal components according to the principle that the variance contribution rate is greater than 85%, and calculate the comprehensive principal component score with the variance contribution rate as the weight, which is the quantitative evaluation value of urban rainfall and flood risk data. The main steps of principal component analysis are [36]:

On the basis of the standardized matrix $X_{11\times 10}$, the correlation matrix $R=\left(r_{jk}\right)$ is calculated.

(3) $$R_{jk}=\frac{\sum_{i=1}^{11}x_{ij}\times x_{ik}}{11-1}\;,\;(j,\;k=1,\;2,\;\ldots ,\;10),$$

Find the eigenvalue $\lambda_{j}$ of the matrix R. According to the principle that the contribution rate of variance is greater than 85% ($\frac{\sum_{j=1}^{m}\lambda_{j}}{\sum_{j=1}^{10}\lambda_{j}}\geq 85\%$), the number of main components *m* is determined. Take the variance contribution rate *t* of each principal component as the weight, multiply by the corresponding principal component *C*, and calculate the comprehensive score. Each comprehensive score is expanded 100 times to get the final evaluation value $y_{1}$, and the $y_{1}$ value of each index is $y_{j}$.

(4) $$y_{j}=\sum_{n=1}^{m}T_{n}C_{n}\times 100,\;(i=1,\;2,\;\ldots ,\;10;\;n=1,2,\;\ldots ,\;m),$$

Calculate the weighted values of 10 indicators in 11 areas based on the value $Y_{1}$. Each area has a corresponding value $Y_{1}$.

(5) $$Y_{1}=y_{j}\times \sum_{j=1}^{10}X_{j},\;(j=1,\;2,\;\ldots ,\;11),$$

#### 3.3.3. The Core Idea of MLWPCA

On the basis of principal component analysis, an extended structural method, multi-layer weighted principal component analysis method is developed. Its core idea is to divide index subsystems based on factor analysis, give importance to each subsystem according to factor score weights. Principal component analysis is performed for each subsystem, and the results of principal component analysis for each subsystem are weighted. The main steps of multi-layer weighted principal component analysis method are as follows: 

Firstly, factor analysis is carried out on the standardized matrix $X_{11\times 10}$. Then, according to the principle that the contribution rate of variance is greater than 80%, to select m main factors. Finally, all factors will be divided into t (*m* = t) subsystems. Each subsystem includes P indexes. 

Secondly, the calculation Equation for the importance of weighting weight of each subsystem is as follows.

(6) $$\omega_{f}=\frac{\sum_{i=1}^{m}\sum_{j=1}^{p}\beta_{ij}\times e_{i}}{\sum_{f=1}^{t}\sum_{i=1}^{m}\sum_{j=1}^{p}\beta_{ij}\times e_{i}}\;(f=1,2,\ldots ,\;t;\;i=1,2,\;\ldots ,\;m;\;j=1,\;2,\;\ldots ,\;p),$$

where $\omega_{f}$ is weights the importance of each subsystem, f is the ordinal number of the system, *i* is the ordinal number of the main factor, *j* is the number of indicators, $\beta_{ij}$ is scoring coefficient for each factor, and $e_{i}$ is the variance contribution rate; usually, the condition is $\sum_{i=1}^{m}e_{i}\geq 80\%$.

Thirdly, each subsystem performs principal component analysis separately. Their steps are the same as the above principal component analysis and the final scores are all increased by 100 times. The calculation method of $y_{2m}$ is the same as Equation (4). Weighted synthesis is performed according to the weighted weight of each subsystem.

(7) $$y_{2}=\sum_{m}^{t}y_{2m}\times T_{m}\;(m=1,2,\;\ldots ,\;t),$$

where $T_{m}$ represents the variance contribution rate of each subsystem after being divided into m indicator subsystems.

Finally, use Equation (5) to calculate the value $Y_{2m}$ and value $Y_{2}$.

## 4. Results and Discussion

### 4.1. The Result of PCA

After standardizing the rainstorm data in Nanjing, the principal component analysis was performed. According to the principle that the cumulative variance contribution rate of the characteristic value was greater than 85%, a total of 3 principal components were selected. Their eigenvalues are 5.824, 1.697, and 1.108. The variance contribution rates are 58.241%, 16.971%, and 11.080%, respectively. Moreover, the overall variance contribution rate is 86.292%. The total variance explained is shown in Table 2. The component matrix of the principal component analysis is shown in Table 3.

Firstly, the indicators in Table 3 are weighted according to the variance contribution rate, and the comprehensive index $Y_{1}$ of the risk assessment of rainstorm disaster in Nanjing is obtained. After calculation, the comprehensive index of each indicator is shown in Table 4. Then, the comprehensive index of urban rainstorm risk of 11 districts in Nanjing in 2018 is calculated through 10 indexes, as shown in Table 5. It can be seen from Table 5 that the range of the comprehensive index $Y_{1}$ is 0.89 ~74.19. The risk level of urban rainstorm disaster decreases with the increase of the value $Y_{1}$. As shown in Table 6, the relationship between the comprehensive index $Y_{1}$ and the risk level of urban rainstorm disaster is based on Li’s [37] classification method. When the range of $Y_{1}$ is 0.89~28.4, the risk level is high. When the range of $Y_{1}$ is 28.41~39.13, the risk level is medium high. When the range of $Y_{1}$ is 39.13~49.86, the risk level is medium low. However, the risk level is low when the range of $Y_{1}$ is 49.86~74.19. The results are listed in Table 4.

**Figure 2:**
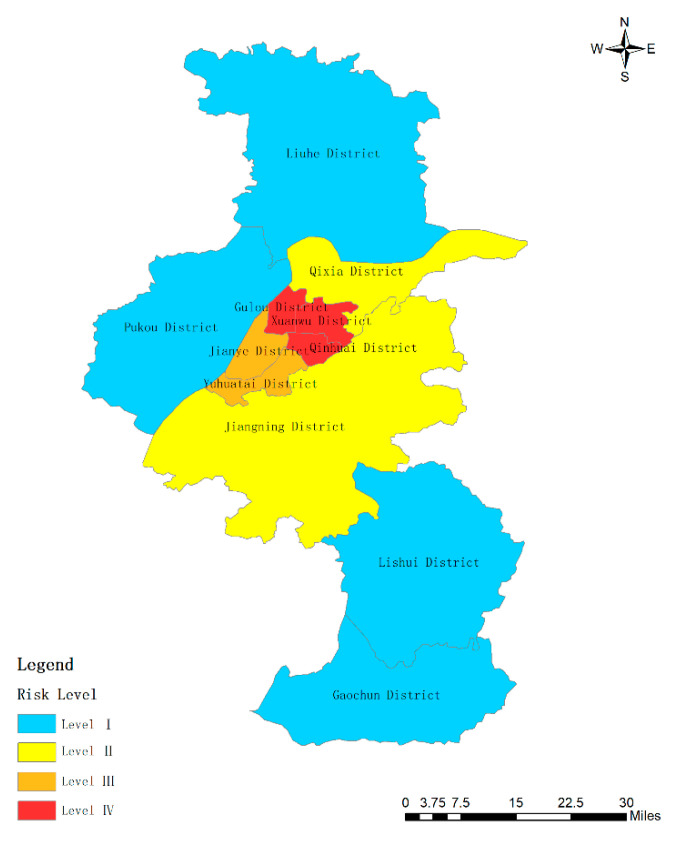
The levels of urban rainstorm risk levels in Nanjing.

By comparing Table 5 and Table 7, there are three districts (Qinhuai District, Gulou District, and Xuanwu) in Nanjing that have a high level of urban rainstorm risk, while two districts (Jianye District, and Yuhuatai District) enjoy medium high risk. There are only two districts with medium low risk, which are Jiangning District and Qixia District. There are four low-risk areas, including Pukou District, Liuhe District, Lishui District, and Gaochun District. The spatial distribution of rainstorm risk areas was described using ArcGIS software (Figure 2). The China Meteorological Administration issued Order No. 16 in 2007 “Measures for the Issuance and Propagation of Early Warning Signals of Meteorological Disasters” [38], which successively used blue, yellow, orange, and red to indicate the risk from low to high. According to the spatial distribution of risk level index ($Y_{1}$) in Figure 2, it can be seen that the distribution of risk levels is generally blue in the north and south, yellow in the middle, and red in a small part. The central part of Nanjing has a high risk of rainstorm disaster. These areas are mainly downtowns. They are relatively prosperous as well. Population, buildings, subway, etc. are very dense. Rainstorm disaster will bring more economic losses and casualties to these areas. The risk of rainstorm in the northern and southern parts of Nanjing is at a medium low level and low level. This is because these places are relatively remote, which are filled with farmland, trees, and lakes. Thus, the capacity for flood storage of these districts is strong. Moreover, the residential population and buildings in these areas are relatively scattered. The roads are wide, and there are more river branches. Thus, the rainstorm disaster will do little harm to people and property.

### 4.2. The Result of MLWPCA

In order to further improve the accuracy of results of urban rainstorm risk assessment, this study further analyzes 10 indicators by using multi-layer weighted principal component analysis method. According to the selection principle of the cumulative variance contribution rate greater than 80% and the eigenvalue greater than 1 through factor analysis, their variance contribution rates are 58.241%, 16.971%, and 11.080%, respectively. The results are listed in Table 8. To make the classification clearer, the maximum orthogonal rotation method was used. Its component score coefficient matrix is listed in Table 9. 

The level of the loading values of each indicator is divided into 3 subsystems. In the first factor, the loading values of $X_{1}$,$\;X_{2}$, $X_{3}$, and $X_{10}$ are high, so these indicators are divided into subsystem 1. $y_{21}$ represents the coefficient of indictors of subsystem 1. In the second factor, the loading values of $X_{4}$, $X_{5}$, and $X_{6}$ is relatively high, so these indicators are divided into subsystem 2. $y_{22}$ represents the coefficient of indictors of subsystem 2. In the third factor, the loading values of $X_{7}$, $X_{8}$, and $X_{9}$ is also relatively high, so these indicators are divided into subsystem 3 (differences of individual data should refer to actual conditions).$\;y_{23}$ represents the coefficient of indictors of subsystem 3. 

**Figure 3:**
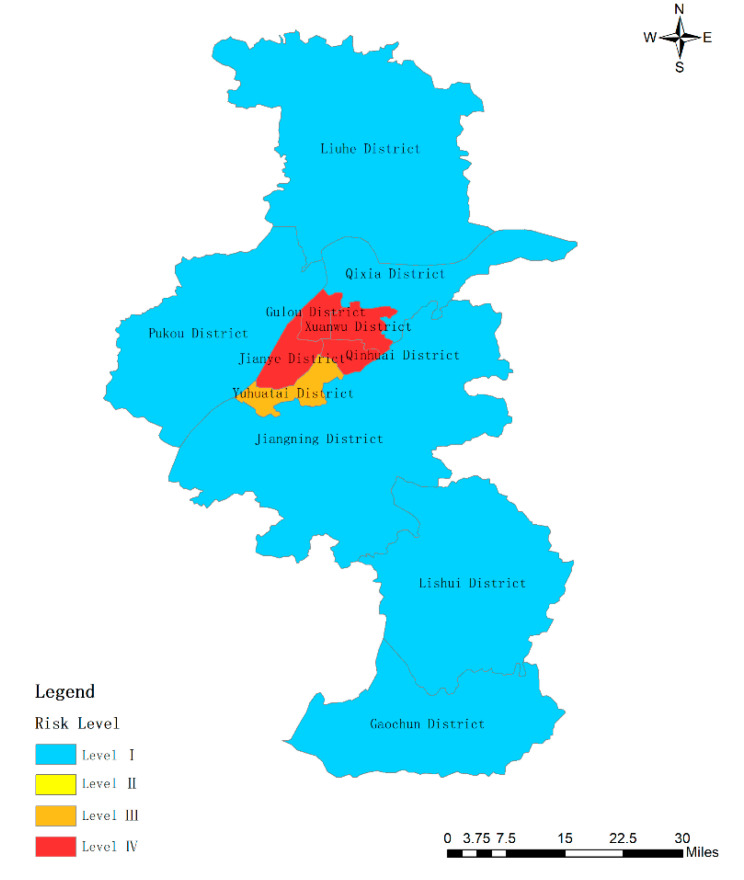
Risk level of subsystem $Y_{21}$ in Nanjing.

**Figure 4:**
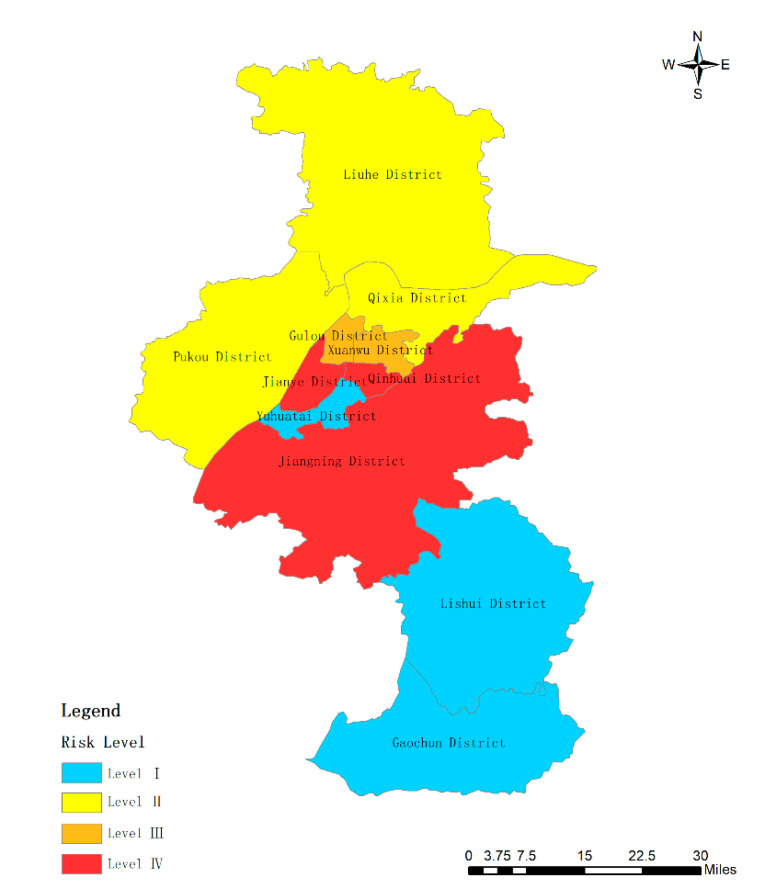
Risk level of subsystem *Y*_22_ in Nanjing.

**Figure 5:**
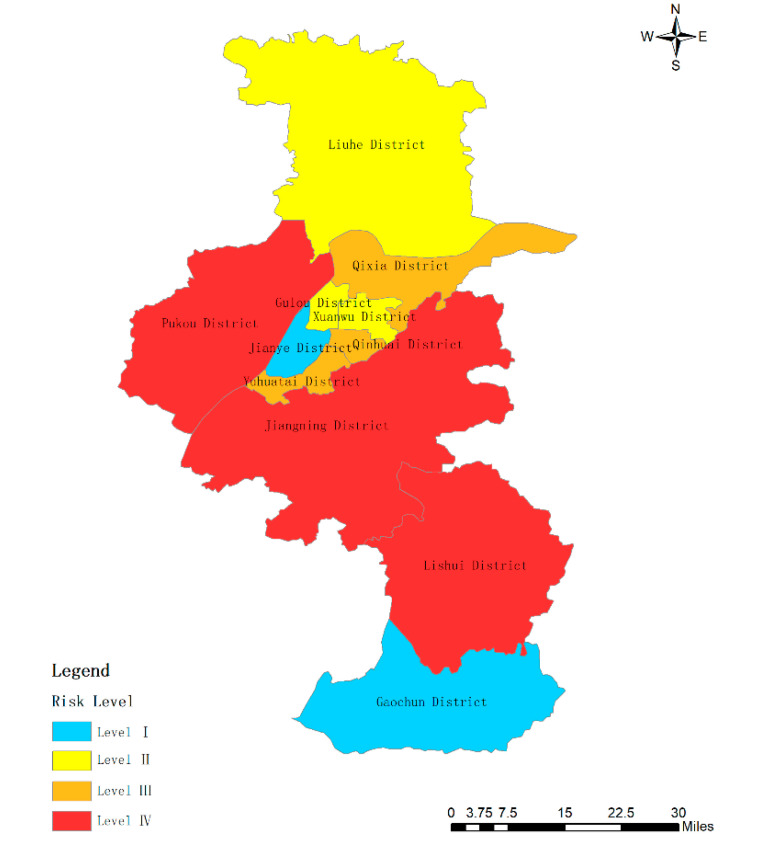
Risk level of subsystem $Y_{23}$ in Nanjing.

**Figure 6:**
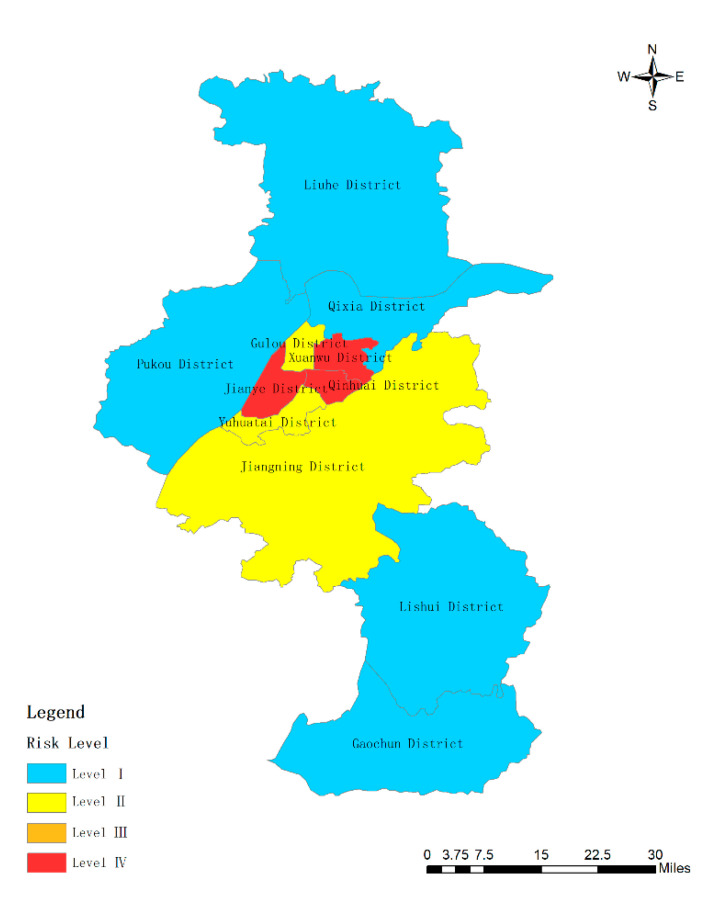
Risk level of subsystem $Y_{2}$ in Nanjing.

Then, the weight of each subsystem can be calculated according to Equation (6) as the basis for evaluating the importance of the subsystems. After calculation, the weights of subsystems 1, 2, and 3 are 0.54, 0.44, and 0.02, respectively. The principal component analysis is performed on the three subsystems, and the variance contribution rates of subsystems 1, 2, and 3 are 90.474%, 87.639% (there are two principal components in subsystem 2, the variance contribution rate of the first factor is 54.539%), and 93.294%, respectively. Moreover, three composite indexes can be obtained including $\;y_{21}$, $y_{22}$, and $y_{23}\;$ based on the variance contribution rate of the principal components in each subsystem. The weight of three subsystems can get $y_{2}$_._ These coefficients of indicators are listed in Table 10. Finally, the study can use Equation (5) to calculate the Value including $Y_{21}$, $Y_{22}$, $Y_{23}$, and $Y_{2}$. Table 11 shows the coefficient of indictors in Nanjing cities. Figure 3, Figure 4 and Figure 5 show the results of the three subsystems. The spatial distribution of urban rainstorm risk in Nanjing cities is shown in Figure 6.

According to the classification method of urban rainstorm risk in Table 6, the risk situation of different districts in different subsystems can be obtained, combining with the composite index of urban rainstorm risk index in Nanjing’s 11 districts in Table 11. All results are listed in Table 12. Some conclusions can be achieved from the comparison of the two sets of data in Table 11 and Table 12.

(1) Subsystem $Y_{21}$ (GDP of per land, population density, building density, subway station density): The composite index ranges from 4.9 to 94.97. As shown in Figure 3, when the composite index ranges from 4.9 to 48.94, high risk appears. All of Gulou District, Qinhuai District, Xuanwu District, and Jianye District are high-risk areas of urban rainstorm disaster. When the composite index ranges from 48.94 to 65.00, medium high risk appears. The risk level in Yuhuatai district is medium high. There is no area where the risk level is medium high. The others are at low risk. Among them, the low-risk areas account for 54.55%. The larger the GDP of per land, population density, building density, and subway station density, it shows that the more people flow, the more intensive the public transportation, and the more developed the economy. Therefore, the rainstorm disaster will bring more economic losses and casualties in these areas. By comparing the original data, the indicator values of four high-risk districts are very high, even more than ten times comparing with low-risk districts. The index here refers to GDP of per land, population density, building density, subway station density. Due to the principle called “smaller, better”, the scores of these four districts are low. The lower the comprehensive score is, the higher the risk and the more vulnerable the hazard-affected body is. 

(2) Subsystem $Y_{22}$ (vegetation coverage, percentage of water area, drainage network density): The composite index ranges from −15.27 to 86.11. As shown in Figure 4, when the composite index ranges from −15.27 to 29.00, high risk appears. The color of Qinhuai District, Jianye District, and Jiangning District is red, so the disaster-pregnant environment in these areas is easier to damage. Although the drainage pipe network density of Qinhuai District is relatively large from the original data, its vegetation coverage and water area percentage are the smallest. This leads to the most risk of rainstorm disaster in Qinhuai District. The vegetation coverage and water area percentage in Jiangning District are both medium levels, but the density of drainage network is small causing rainwater to be discharged in a timely manner. This leads to a risk of water accumulation. Jianye District has many office buildings for commercial offices with less greening. This also causes water accumulation. Thus, the risk level of urban rainstorm disaster in Jiangning District and Jianye District is also high. When the composite index ranges from 29.00 to 42.61, medium high risk appears. The color of Xuanwu District and Gulou District is orange. Although Xuanwu District and Gulou District are located in the center of the city, the rainstorm disaster risk in these two areas is medium high due to the higher values of vegetation coverage, percentage of water area, and drainage network density. The color of Pukou District, Qixia District, and Liuhe District is yellow as their indicators values in the subsystem $Y_{22}$ is medium low level. When the composite index ranges from 56.21 to 86.11, low risk appears. The color of Yuhuatai District, Gaochun District, and Lishui District is blue. The main reason for the low risk of Yuhuatai District is the high density of drainage network, and for the low risk of Gaochun District and Lishui District is the smallest building density in Nanjing. It can be seen that the density of drainage network is related closely to reduce the urban rainstorm disaster.

(3) Subsystem $Y_{23}$ (24-h maximum rainfall, number of rainstorm days, total rainfall): The composite index ranges from −23.23 to 79.63. These three indicators indicate the danger of hazard factors. The more rainfall there is, the longer days that the rainfall lasts and the greater the risk of urban rainstorm disaster is. According to the observation of the original data, the rainfall in 11 districts of Nanjing is relative, and there is a lot of rainfall in each district. Therefore, there is no obvious difference in the impact of disaster-causing factors on 11 districts of Nanjing. Thus, the hazard factors will not cause obvious differences in the risk of urban rainstorm in Nanjing city. When the composite index ranges from −23.23 to 6.87, high risk appears. Relatively speaking, Pukou District, Jiangning District, and Lishui District have higher risk of rainstorm disaster. The result is shown in Figure 5. However, the weight of the subsystem $Y_{23}$ in the three subsystems is very small, the three index values including 24-h maximum rainfall, rainstorm days, and total rainfall have little effect on the risk of urban rainstorm disaster in Nanjing.

(4) A composite index $Y_{2}$ is weighted by three subsystems. The composite index ranges from 0.97 to 90.59. When the composite index ranges from 0.97 to 40.73, high risk appears. When the composite index ranges from 40.73 to 54.31, medium high risk appears. When the composite index ranges from 54.31 to 67.89, medium low risk appears. When the composite index ranges from 67.89 to 90.59, low risk appears. Qinhuai District, Gulou District, Jianye District, and Xuanwu District are all high-risk areas for urban rainstorm risk. Yuhuatai District and Jiangning District belong to medium low rainstorm risk zones, while the other five areas including Qixia District, Pukou District, Liuhe District, Lishui District, and Gaochun District are all low-risk zones. There is no yellow area in Figure 6. Thus, there is no low-risk district by using multi-layer weighted principal component analysis method. As seen from the figure, the results of Figure 6 is similar to the results of Figure 3 and Figure 4 and is completely different from Figure 4. This is because the weight ratio of $Y_{21}$ and $Y_{22}$ is 98%, and the weight of $Y_{23}$ is only 0.02. Therefore, the distribution of urban rainstorm risk level in Nanjing is mainly affected by the hazard-affected body and the disaster-pregnant environment. Furthermore, it is obvious from the figure that developed areas are more vulnerable to the impact of rainstorm disasters, which is consistent with the research of some scholars [39].

At the same time, the results of principal component analysis are basically consistent with multi-layer weighted principal component analysis method. In both methods, the color of Pukou District, Liuhe District, Lishui District, and Gaochun District are all Blue, which means they are low-risk areas. The color of Jiangning District and Qixia District are both yellow, which means they are medium low-risk areas. The color of Xuanwu District and Qinhuai District are both red, which means they are high-risk areas. There are some differences between the two methods. In principal component analysis, the color of Gulou District is red, which means it is high-risk area. The color of Jianye District and Yuhuatai District is orange, which means they are medium high-risk areas. In multi-layer weighted principal component analysis method, the color of Gulou District and Yuhuatai District is yellow, which means they are medium low-risk areas. The color of Jianye District is red, which means it is high-risk area. Because in the subsystem 2 the values of vegetation coverage, percentage of water area, and drainage network density are relatively high, that is, the disaster-pregnant environment is not easy to be damaged. Jianye District is widely filled with office buildings for commercial offices while its greening is less. Thus, it is more likely to cause water accumulation. The environment is more susceptible to damage. All in all, multi-layer weighted principal component analysis method not only makes the results objective but also judges the risk status of each subsystem in the urban rainstorm disaster risk.

### 4.3. Regulation Countermeasures

According to the results of the distribution of rainstorm disaster risk in Nanjing, the government should pay attention to the prevention and control of rainstorm disaster. From the perspective of modern risk management, combined with the experience of rainstorm risk management in other cities, this paper explores the implementation ways of rainstorm disaster risk management in Nanjing through engineering measures and non-engineering measures. In addition, the term “sponge city” is based on the reference and absorption of foreign urban rainwater flood control ideas. The United States, Japan, and other developed countries have achieved good results in rainwater resource utilization and management [40,41]. China is also doing pilot research on “sponge city” [42]. Therefore, this paper puts forward the following suggestions for rainstorm control and disaster reduction in Nanjing from three aspects: engineering measures, non-engineering measures, and “sponge city” construction. 

Firstly, appropriate engineering control measures should be taken according to local conditions to reduce the risk of rainstorm disaster. The government should strengthen the construction and maintenance of water conservancy projects, improve the system of rainstorm control works, and raise the rainstorm control standards. Rivers and lakes such as Qinhuai River and Lishui River should be monitored and cleaned up in time. In order to improve the drainage capacity of the drainage facilities in Nanjing, the underground pipe network must be overhauled in real-time. The artificial reservoir and water storage lake in the main urban area of Nanjing are maintained in real-time to improve their water storage capacity. In order to ensure the regulation and storage capacity of the reservoir, real-time monitoring should be carried out for the reservoir water regime in the suburbs. It is necessary for the main areas of Nanjing to regularly repair green parks such as Xuanwu Park to improve the infiltration capacity of the ground. At the same time, when the real estate firm develops the residential areas, the proportion of permeable ground should be more than 80%, so as to reduce the risk of urban rainstorm disaster. The government must also control unsuitable land use and balance economic development and risk management [43]. Water conservancy department should do a good job in monitoring rainstorm [44]. Of course, the rainstorm disaster in Nanjing will also be affected by the surrounding cities, especially the upstream cities. Therefore, it is necessary for Nanjing to cooperate with surrounding cities to control rainstorm disasters and build a beautiful and safe ecosystem.

Secondly, the government should improve the emergency management mechanism and implement the flood prevention responsibility mechanism. When there is a rainstorm, relevant departments should respond quickly, reasonably arrange relevant personnel to enter the disaster site for rescue, and reasonably arrange the distribution of materials to ensure the smooth transportation channel of materials and personnel. It is necessary to carry out comprehensive and systematic propaganda on the prevention and reduction of urban rain flood disasters, so as to improve the people’s awareness of disaster prevention and reduction. In some areas where rainstorms and floods often occur, people’s skills of avoiding disasters can be enhanced through practical exercises.

Finally, due to the abundant precipitation in summer, Nanjing can learn from the experience of “sponge city” to reduce the risk of waterlogging. Through the collection of rainwater, rainwater storage, slow infiltration, purification of water, and when necessary, the rainwater stored in the city can be discharged or utilized. With the help of natural forces, the rainwater from the source can be collected, saved, purified, and discharged nearby, so as to realize the natural migration of rainwater in the city. Combined with the characteristics of Nanjing, we should do a good job in the construction of four major technical projects: the diversion project of rainwater and sewage, the road treatment project, the rainwater storage project, and the optimization project of water regulation and storage [45].

## 5. Conclusions

Nanjing is affected by rainstorm disasters every year. The focus of this paper is to understand the specific risk distribution of rainstorm disaster and to find out the causes of disasters in each district. Firstly, the multi-layer weighted principal component analysis method constructed in this paper is based on factor analysis. all indicators were divided into several subsystems. Then, in view of the importance of the subsystem, weighting is carried out by factor score weight. Multi-layer weighted principal component analysis method can better highlight the weight of different indicators in the risk assessment of urban rainstorm disaster than the principal component analysis Method. This method makes it easier to derive the dominant factors for urban rainstorm disaster risk levels. It can also avoid the disadvantages brought by subjective weighting and make the evaluation results more objective and differentiated. The results showed that the dominant factors in Nanjing cities are the hazard-affected body and the disaster-pregnant environment.

Secondly, during the risk assessment of urban rainstorm disaster process in Nanjing, the results of the risk distribution based on the multi-layer weighted principal component analysis method are basically consistent with those of the traditional principal component analysis Method. However, the results with the multi-layer weighted principal component analysis method are more targeted and distinguishable. In Nanjing city, there is little difference between the regions of precipitation and continuous precipitation days, so the weight of subsystem 3 is small in the risk assessment system of urban rainstorm disaster. Moreover, the vulnerability of hazard-affected body and the fragility of disaster-pregnant environment are more different in terms of the urban rainstorm disaster risk. So, they have bigger proportions. From the data analysis results, the multi-layer weighted principal component analysis method is more reasonable. It is worth popularizing as a method of the risk assessment of urban rainstorm disaster. According to the research results, the high-risk areas in Nanjing cities is mainly concentrated in the prosperous areas such as Gulou District, Xuanwu District, Qinhuai District, and Jianye District. However, the reasons for each regional risk are different. The high-risk areas including Gulou District, Jianye District, Qinhuai District, and Xuanwu District are due to the vulnerable hazard-affected body and fragile disaster-pregnant environment. The high risk of rainstorm disaster in Pukou District, Jiangning District, and Lishui District is that the hazard factors are more dangerous and the disaster-pregnant environment is more fragile. Thus, the government must take appropriate measures to reduce the risk of rainstorm disaster in each district. 

Finally, this article focuses on the risk distribution of rainstorm disaster in space and does not do time research, so there are some limitations. In the next study, we will expand the study area to study the rainstorm risk of 26 cities in the Yangtze River Delta from the perspective of time and space. It can provide reference and suggestions for relevant departments to deal with rainstorm disasters. Furthermore, this method can also be applied to other fields, such as water quality assessment and security assessment of water-energy-food system. 

## Figures and Tables

**Table 1:** Statistics of the rainstorm-affected population, deaths, missing persons, and direct economic losses from 2010 to 2018.

Year	Rainstorm-Affected Population (Ten Thousand People)	Death Population (One Person)	Missing Population (One Person)	Direct Economic Losses (Billion Yuan)
2010	21,084.68	3222	1003	3745.43
2011	8941.70	519	121	1301.27
2012	12,367.11	673	159	2675.32
2013	11,974.27	775	374	3155.74
2014	7381.82	486	91	1573.55
2015	7640.85	319	81	1660.75
2016	10,095.41	686	207	3643.26
2017	5514.90	316	39	2142.53
2018	5576.55	187	32	1615.47

**Table 2:** Total variance explained.

Components	Initial Eigenvalues			Extraction Sums of Squared Loading		
	Total	% of Variance	Cumulative %	Total	% of Variance	Cumulative %
1	5.824	58.241	58.241	5.824	58.241	58.241
2	1.697	16.971	75.212	1.697	16.971	75.212
3	1.108	11.080	86.292	1.108	11.080	86.292
4	0.732	7.325	93.616			
5	0.293	2.932	96.548			
6	0.203	2.027	98.576			
7	0.104	1.041	99.616			
8	0.029	0.292	99.909			
9	0.009	0.089	99.998			
10	0.000	0.002	100.000			

**Table 3:** Component score coefficient matrix.

Indicators	The Meanings of Indicators	Component		
		1	2	3
$X_{1}$	GDP of per land (billion yuan/km^2^)	0.159	0.018	−0.046
$X_{2}$	population density (person/km^2^)	0.156	0.080	−0.143
$X_{3}$	building density (%)	0.167	−0.023	−0.119
$X_{4}$	percentage of vegetation coverage (%)	0.089	0.396	0.020
$X_{5}$	percentage of water area (%)	0.153	0.027	0.071
$X_{6}$	drainage network density (km/km^2^)	−0.014	−0.197	0.826
$X_{7}$	24-h maximum rainfall (mm)	0.088	−0.459	−0.077
$X_{8}$	rainstorm days (days)	0.050	0.391	0.384
$X_{9}$	total rainfall (mm)	−0.162	0.124	−0.127
$X_{10}$	subway station density (one/100 km^2^)	0.161	−0.079	0.094

**Table 4:** Coefficient of indicators of $y_{1}$ index.

Indicators	$X_{1}$	$X_{2}$	$X_{3}$	$X_{4}$	$X_{5}$	$X_{6}$	$X_{7}$	$X_{8}$	$X_{9}$	$X_{10}$
$y_{1}$	9.06	8.86	8.02	12.13	10.16	5.00	−3.52	13.80	−8.74	9.08

**Table 5:** Risk classification of $Y_{1}$ composite index of urban rainstorm risk in Nanjing cities in 2008.

Districts	Xuanwu	Qianhuai	Jianye	Gulou	Pukou	Qixia
$Y_{1}$	19.11	0.89	31.86	9.01	52.08	45.45
**Districts**	**Yuhuatai**	**Jiangning**	**Liuhe**	**Lishui**	**Gaochun**	
$Y_{1}$	37.28	42.77	60.48	57.35	74.19	

**Table 6:** Urban rainstorm risk and assessment index.

The Range of Composite Index	Risk Level
Minimum value of composite index~average value—Half of the standard deviation	High (Ⅳ)
Average value—Half of the standard deviation~average value	Medium high (Ⅲ)
Average value~average value + Half of the standard deviation	Medium low (Ⅱ)
Average value + Half of the standard deviation~Maximum value of composite index	Low (Ⅰ)

**Table 7:** Assessment result.

The Range of Composite Index	Risk Level
0.89~28.41	High (IV)
28.41~39.13	Medium high (III)
39.13~49.86	Medium low (II)
49.86~74.19	Low (I)

**Table 8:** Total variance Explained.

Components	Initial Eigenvalues			Extraction Sums of Squared Loading		
	Total	% of Variance	Cumulative %	Total	% of Variance	Cumulative %
1	5.824	58.241	58.241	5.696	56.964	56.964
2	1.697	16.971	75.212	1.746	17.463	74.426
3	1.108	11.080	86.292	1.187	11.865	86.292

**Table 9:** Rotated component matrix and component score coefficient matrix.

Indicators	The Meanings of Indicators	Rotated Component Matrix			Component Score Coefficient Matrix		
		1	2	3	1	2	3
$X_{1}$	GDP of per land (billion yuan/km^2^)	0.905	0.167	−0.105	0.153	0.028	−0.057
$X_{2}$	population density (person/km^2^)	0.875	0.226	−0.240	0.141	0.052	−0.169
$X_{3}$	building density (%)	0.965	0.082	−0.160	0.170	−0.034	−0.113
$X_{4}$	percentage of vegetation coverage (%)	0.393	0.717	−0.232	0.018	0.388	−0.119
$X_{5}$	percentage of water area (%)	0.868	0.220	0.014	0.145	0.075	0.050
$X_{6}$	drainage network density (km/km^2^)	−0.030	−0.012	0.977	0.013	0.096	0.844
$X_{7}$	24-h maximum rainfall (mm)	0.642	−0.663	0.156	0.168	−0.436	0.077
$X_{8}$	rainstorm days (days)	0.166	0.808	0.162	−0.023	0.501	0.227
$X_{9}$	total rainfall (mm)	−0.967	−0.012	−0.155	−0.181	0.044	−0.153
$X_{10}$	subway station density (one/100 km^2^)	0.947	0.069	0.096	0.172	−0.014	0.107

**Table 10:** Coefficient of indictors.

Indictors	X_1_	X_2_	X_3_	X_4_	X_5_	X_6_	X_7_	X_8_	X_9_	X_10_
$y_{21}$	24.16	23.80	24.34	0	0	0	0	0	0	22.71
$y_{22}$	0	0	0	49.20	47.07	−15.27	0	0	0	0
$y_{23}$	0	0	0	0	0	0	−38.69	77.71	15.69	0
$y_{2}$	13.05	12.85	13.14	21.65	20.72	−6.72	−0.77	1.55	0.31	12.26

**Table 11:** Coefficient of indictors in Nanjing cities.

Districts	Xuanwu	Qianhuai	Jianye	Gulou	Pukou	Qixia
$Y_{21}$	41.68	13.96	43.70	4.90	89.46	84.09
$Y_{22}$	33.03	−15.27	16.58	31.88	49.97	54.86
$Y_{23}$	37.34	7.75	79.63	25.37	0.82	21.29
$Y_{2}$	37.78	0.97	32.48	17.18	70.32	69.97
**Districts**	**Yuhuatai**	**Jiangning**	**Liuhe**	**Lishui**	**Gaochun**	
$Y_{21}$	64.84	90.37	92.34	94.97	94.71	
$Y_{22}$	60.13	22.38	56.16	72.85	86.11	
$Y_{23}$	10.42	−19.45	34.74	−23.23	77.91	
$Y_{2}$	61.68	58.26	75.27	82.88	90.59	

**Table 12:** Assessment result.

Subsystem	The Range of Composite Index	Risk Level
$Y_{21}$	4.9~48.94	High (Ⅳ)
	48.94~65.00	Medium high (Ⅲ)
	65.00~81.06	Medium low (Ⅱ)
	81.06~94.97	Low (Ⅰ)
$Y_{22}$	−15.27~29.00	High (Ⅳ)
	29.00~42.61	Medium high (Ⅲ)
	42.61~56.21	Medium low (Ⅱ)
	56.21~86.11	Low (Ⅰ)
$Y_{23}$	−23.23~6.87	High (Ⅳ)
	6.87~22.96	Medium high (Ⅲ)
	22.96~39.06	Medium low (Ⅱ)
	39.06~79.63	Low (Ⅰ)
$Y_{2}$	0.97~40.73	High (Ⅳ)
	40.73~54.31	Medium high (Ⅲ)
	54.31~67.89	Medium low (Ⅱ)
	67.89~90.59	Low (Ⅰ)
